# Gallic Acid Reduces Blood Pressure and Attenuates Oxidative Stress and Cardiac Hypertrophy in Spontaneously Hypertensive Rats

**DOI:** 10.1038/s41598-017-15925-1

**Published:** 2017-11-15

**Authors:** Li Jin, Zhe Hao Piao, Simei Sun, Bin Liu, Gwi Ran Kim, Young Mi Seok, Ming Quan Lin, Yuhee Ryu, Sin Young Choi, Hae Jin Kee, Myung Ho Jeong

**Affiliations:** 1 Heart Research Center of Chonnam National University Hospital, Gwangju, 61469 Republic of Korea; 2 Jilin Hospital Affiliated with Jilin University, Jilin, China; 3 Department of Cardiology, The Second Hospital of Jilin University, Changchun, Jilin, 130041 China; 4 National Development Institute of Korean Medicine, Hwarang-ro, Gyeongsan-si, Gyeongsangbuk-do, Republic of Korea; 5 Yanbian University Hospital, 1327 Juzi road, Jilin Yanbian China

## Abstract

Gallic acid (GA) has been reported to have beneficial effects on cancer, vascular calcification, and diabetes-induced myocardial dysfunction. We hypothesized that GA controls hypertension via oxidative stress response regulation in an animal model for essential hypertension. Spontaneously hypertensive rats (SHRs) were administered GA for 16 weeks. GA treatment lowered elevated systolic blood pressure in SHRs through the inhibition of vascular contractility and components of the renin-angiotensin II system. In addition, GA administration reduced aortic wall thickness and body weight in SHRs. In SHRs, GA attenuated left ventricular hypertrophy and reduced the expression of cardiac-specific transcription factors. NADPH oxidase 2 (Nox2) and GATA4 mRNA expression was induced in SHR hearts and angiotensin II-treated H9c2 cells; this expression was downregulated by GA treatment. Nox2 promoter activity was increased by the synergistic action of GATA4 and Nkx2-5. GA seems to regulate oxidative stress by inhibiting the DNA binding activity of GATA4 in the rat Nox2 promoter. GA reduced the GATA4-induced Nox activity in SHRs and angiotensin II-treated H9c2 cells. GA administration reduced the elevation of malondialdehyde levels in heart tissue obtained from SHRs. These findings suggest that GA is a potential therapeutic agent for treating cardiac hypertrophy and oxidative stress in SHRs.

## Introduction

Chronic hypertension adversely affects critical organs, such as brain, eyes, heart, and kidneys^1^. Hypertension is the most important determinant for left ventricular hypertrophy (LVH) and is a major risk factor for cardiovascular diseases^2^. Echocardiography is the gold standard for evaluating left ventricular (LV) mass, cardiac function, wall thickness, and chamber size^3^. LVH prevalence varies with the severity of hypertension^4^.

For experiments on animals with hypertension, spontaneously hypertensive rats (SHRs) are widely used models that exhibit essential hypertension similar to that in humans^5^. Cardiac hypertrophy manifests at about 12 weeks of age in SHRs^6^. Various pathophysiologic factors contribute to hypertension development. For example, the activation of the renin-angiotensin-aldosterone system (RAAS), the production of vasoconstrictors, endothelial dysfunction, sodium intake, and oxidative stress play critical roles in hypertension^7^. In the human INTERSALT study, body mass index was significantly related to hypertension in men and women^8^. Obesity-induced LVH may be associated with concentric LV remodeling^9,10^. In the RAAS, angiotensin II has been reported to act on angiotensin II receptor (AT1) and elevate blood pressure^11^. In addition, angiotensin II induces cardiac and vascular cell hypertrophy, as well as hyperplasia^12^.

Cardiac hypertrophy and cardiac remodeling is associated with oxidative stress^13^. The nicotinamide adenine dinucleotide phosphate (NADPH) oxidase is a membrane-bound enzyme. NADPH oxidase (Nox) is composed of seven isoforms, including Nox1, Nox2, Nox3, Nox4, Nox5, Duox1, and Duox2^14^. Nox family is a multicomponent enzyme complex with two membrane-bound subunits (Nox and p22phox) and regulatory subunits. Under pathological conditions, the regulatory subunits, including p40phox, p47phox, p67phox, and Rac-1, translocate from the cytosol to the plasma membrane^15^. Nox produces reactive oxygen species (ROS), in particular, superoxide anions and hydrogen peroxide^16^. ROS have been reported to play an important role in the pathophysiology of hypertension^17^. The activities of Nox1, Nox2, and Nox5 cause adverse effects, such as endothelial dysfunction, inflammation, and apoptosis, whereas Nox4 plays a protective role in the vasculature^18^. In hypertension, oxidative stress promotes endothelial dysfunction, inflammation, and vascular remodeling, as well as fibrosis, hypertrophy, and apoptosis^19–21^.

Although several efforts have been made to develop a novel antihypertensive drug, the number of patients with hypertension is still uncontrolled. Recently, natural compounds derived from plants or fruits have been shown to have beneficial or protective effects in various pathological diseases. Gallic acid (GA) is a polyphenolic compound found in grapes, mangoes, walnuts, green tea, and wine. It has been reported to have anti-diabetic, anti-bacterial, anti-inflammatory, anti-angiogenic, anti-oxidant, and anti-cancer activity^22–26^. Furthermore, it has been shown to have neuroprotective effects in rats^27^. GA prevents the changes in the activities of cardiac marker enzymes in isoproterenol-induced myocardial infarction rats^28^. GA attenuates vascular calcification through the BMP2-Smad1/5/8 signaling pathway suppression^29^, suggesting that GA may have a protective role in vascular diseases.

The effects of GA and the regulatory mechanisms by which GA regulates blood pressure in essential hypertension are unknown. We investigated the effects of GA in SHR hearts by assessing LVH, oxidative stress-related gene expression, and ROS and Nox activity.

## Materials and Methods

### Animals and Blood Pressure

All animal procedures were approved by the Animal Experimental Committee of Chonnam National University Medical School (CNU IACUC-H-2014-52) and carried out in accordance with the Guide for the Care and Use of Laboratory Animals (US National Institutes of Health Publications, 8^th^ edition, 2011). Wistar-Kyoto (WKY, 4-week-old males, n = 16) rats and spontaneously hypertensive rats (SHRs, 4-week-old males, n = 16), were purchased from SLC Co. (Shizuoka, Japan).

Rats were divided into three groups: WKY, SHR, and SHR+GA. WKY was used as the control. GA (G7384) was purchased from Sigma Life Science (St. Louis, MO, USA). The purity of GA was more than 97.5%. GA was dissolved in tap water to obtain a final concentration of 1% (1 g/100 mL). In SHRs, GA (1%) was administered from 8 till 24 weeks of age. On average, SHRs drank 320 mg of gallic acid per day. Animals were sacrificed at 24 weeks of age.

Blood pressures were measured as described previously^30^. Briefly, systolic blood pressure was measured in awake rats by the tail-cuff method (Visitech Systems, BP-2000) and the average of at least 5 readings was calculated. Blood pressure and body weight was determined at 8, 12, and 24 weeks of age.

### Echocardiography

Echocardiography was performed, as described previously^31^. Two-dimensionally guided left ventricle (LV) M-mode images at the papillary muscle level, were obtained from the parasternal short axis view. LV interventricular septal thicknesses, and posterior wall thicknesses at the end of diastole and systole were measured from the M-mode images. LV fractional shortening (FS) was calculated as: FS = (LV end-systolic diameter [LVESD] − LV end-diastolic diameter [LVEDD])/LVEDD × 100).

### H&E staining

Heart tissues were fixed in 4% paraformaldehyde at room temperature, embedded in paraffin, and cut into 3-µm thick sections. To identify myocardium status, hematoxylin and eosin (H&E) staining was performed. Sections were deparaffinized with xylene and rehydrated with graded alcohol. The slides were incubated with Gill’s hematoxylin V (Muto, Tokyo, Japan) for 5 min and then washed with tap water. Then, they were incubated with 0.3% HCl for 5–7 min, dipped in Scott’s Bluing solution, and washed with tap water. The cytoplasm was stained with eosin Y for 2–3 min, and the slides were dehydrated and mounted using mounting solution (Canada balsam: xylene = 6:4).

### Immunohistochemistry and arterial wall morphology

For immunohistochemistry, the aorta sections were deparaffinized and pretreated for heat-induced antigen retrieval with citrate buffer. After blocking with 1% bovine serum albumin (BSA), the tissue sections were incubated with anti-SMA antibody (1:800; Santa Cruz, sc-130617) using the LSAB2 system-horseradish peroxidase (HRP) kit (DAKO, USA). A biotinylated secondary antibody (1:400) was added, followed by incubation with Vectastain ABC reagent and treatment with the peroxidase substrate, 3,3ʹ-diaminobenzidine (DAB), until the desired staining intensity was obtained. Finally, the tissue samples were counterstained and mounted. The arterial wall thickness was analyzed using ImageScope (Leica Biosystems, USA).

### Isometric tension measurement

Male Sprague-Dawley rats were purchased from Orient Bio (Gyeonggi-do, South Korea). Thoracic aortas and mesenteric arteries were excised and immersed in ice-cold, modified Krebs solution (in mM: NaCl 115, KCl 4.7, CaCl_2_ 2.5, MgCl_2_ 1.2, NaHCO_3_ 25, KH_2_PO4 1.2, and dextrose 10). Mesenteric arteries were the secondary branches of the main mesenteric trunk. The aortas and mesenteric arteries were cleaned of all connective tissue, soaked in Krebs-bicarbonate solution, and cut into four ring segments (3.5 mm in length), as described previously^32,33^. Some rings were denuded of endothelium by gently rubbing the internal surface with a forcep edge. Each aortic ring was suspended in a water-jacketed organ bath (6 ml) maintained at 37 °C and aerated with a mixture of 95% O_2_ and 5% CO_2_. Each ring was connected to an isometric force transducer (Danish Myo Technology, Skejbyparken, Aarhus N, Denmark). Rings were stretched to an optimal resting tension of 2.0 g or 1.0 g, which was maintained throughout the experiment. Each ring was equilibrated in the organ bath solution for 90 min before the experiment measuring the contractile response after the addition of 50 mM KCl. To determine the effect of gallic acid (GA) on the maintenance of vascular tension in rat endothelium-intact or endothelium-denuded aortic rings, vascular contractions were induced using the thromboxane A2 agonist, U46619 (30 nM, 20 min). When each contraction reached a plateau, GA was added cumulatively (10–3,000 µM) to elicit vascular relaxation.

### Western blot analysis and antibodies

**Figure 1:**
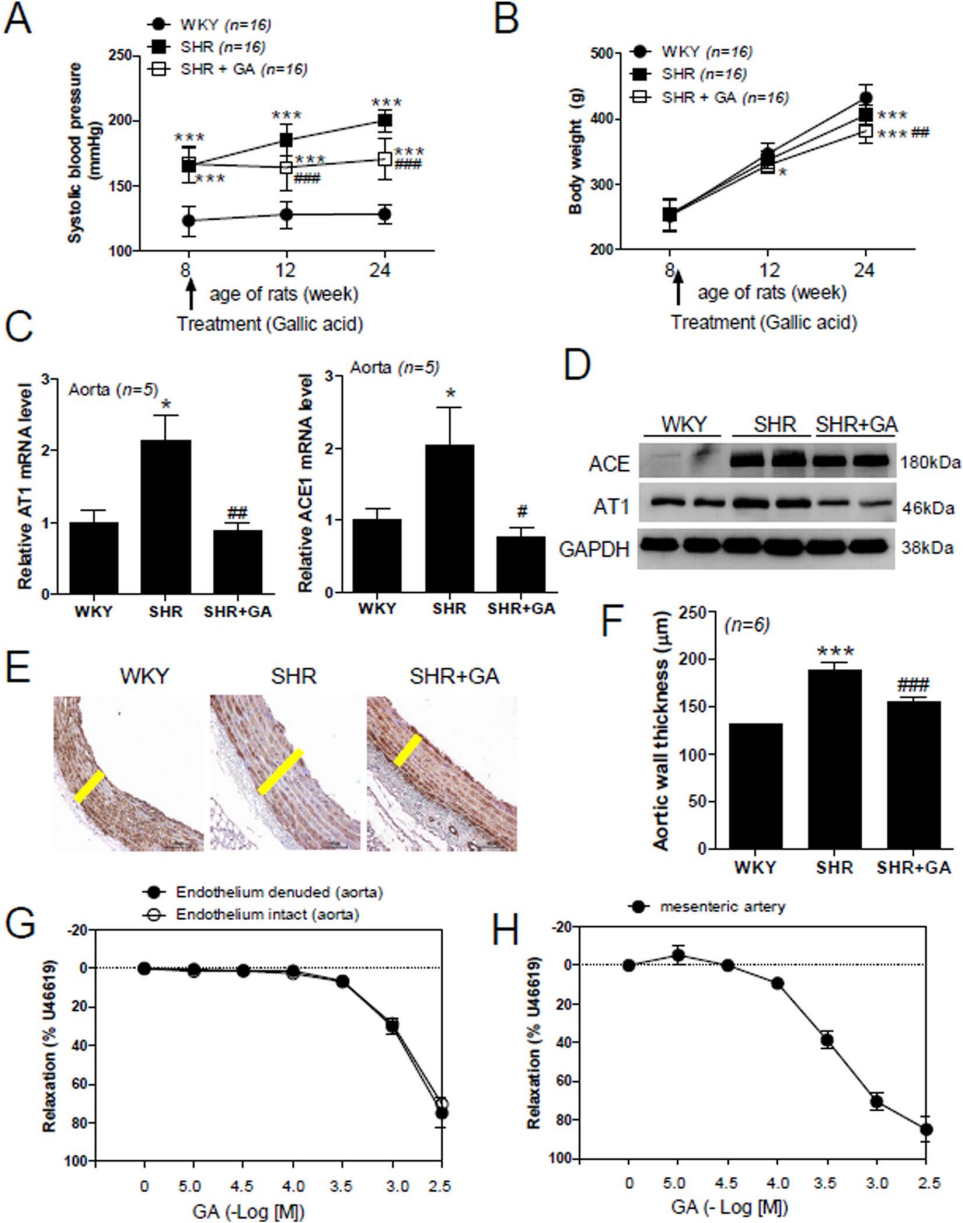
GA reduces blood pressure, vascular remodeling, and contraction in SHRs. (**A**) Systolic blood pressure in the three groups was measured at the indicated time points (n = 16 per group). Rats were administered GA from 8 weeks of age. Statistical analysis was performed by two-way ANOVA. ***P < 0.001 versus control WKY at 8, 12, and 24 weeks. ^###^ *P* < 0.001 versus SHR at 12 and 24 weeks. (**B**) Body weight was compared among the three groups at the indicated time points (n = 16 per group). Statistical analysis was performed by two-way ANOVA. *P < 0.05 and ***P < 0.001 versus WKY at 12 weeks and 24 weeks, respectively. ^##^ *P* < 0.01 versus SHR at 24 weeks. (**C**) Transcript levels of AT1 and ACE1 mRNA were measured by quantitative RT-PCR using rat aortas from the WKY, SHR, and SHR plus GA (SHR+GA) groups. *P < 0.05 versus WKY. ^#^ *P* < 0.05 and ^##^ *P* < 0.01 versus SHR. (**D**) Representative immunoblots. ACE1 and AT1 protein levels were determined by western blot in rat aortas from the WKY, SHR, and SHR+GA groups (n = 6 per group). (**E**) Images of aorta sections immunostained with anti-smooth muscle α-actin (SMA) antibody. Yellow bar indicates the thickness of arterial wall. Scale bar, 100 µm. (**F**) Bar graph indicates aortic wall thickness in each group (n = 6). ***P < 0.001 versus WKY at 24 weeks. ^###^ *P* < 0.001 versus SHR at 24 weeks. (**G**,**H**) Vasorelaxation induced by GA in the aorta (**G**) and mesenteric arteries (**H**) from rats (n = 4). GA was added cumulatively to elicit relaxation in U46619-treated aortic rings and mesenteric arteries. Relaxation is expressed as the percentage of the maximal contractile response to U46619. Open and filled circles represent endothelium-intact and endothelium denuded rat aortic rings, respectively.

Western blots were performed, as described previously^34^. Protein lysates from the left ventricle (for Fig. 4B) or aorta (for Fig. 1D) were prepared with RIPA buffer (150 mM NaCl, 1% Triton X-100, 1% sodium deoxycholate, 50 mM Tris-HCl, pH 7.5, 2 mM ethylenediamine tetraacetic acid (EDTA), 1 mM phenylmethylsulfonylfluoride (PMSF), 1 mM dithiothreitol (DTT), 10 mM Na_3_VO_4_, and 20 mM NaF) containing protease inhibitors. Proteins were separated by 10% SDS-PAGE and then transferred onto polyvinylidene difluoride (PVDF) membranes. The membranes were probed with the indicated antibodies and developed using Immobilon western blotting detection reagents (Millipore, Billerica, MA, USA). Bio-ID software was used to quantify protein expression (Vilber Lourmat, Germany). Anti-GAPDH (sc-32233) and anti-AT1 (sc-1173) antibodies were purchased from Santa Cruz Biotechnology. Anti-Nox1 (GTX103888), -Nox2 (GTX63960), -Nox4 (GTX121929), and -ACE1 (GTX100923) antibodies were purchased from GeneTex Inc. (Irvine, CA, USA).

### RNA isolation and real-time PCR

Total RNA was isolated using TRIzol reagent (Invitrogen Life Technologies), and 1 μg of RNA was used for reverse transcription reaction using TOPscript RT DryMIX (Enzynomics, Daejeon, South Korea). Quantification of mRNA levels was done using SYBR Green PCR kit (Enzynomics, Daejeon, South Korea). Relative expression levels of the indicated genes were compared with that of 18S rRNA, using the 2^−ΔΔct^ method. The primers used are shown in Supplementary Table 1.

### Transfection

H9c2 cells were maintained in Dulbecco’s modified Eagle’s medium (DMEM) supplemented with 10% fetal bovine serum (FBS) plus 1 × antibiotic-antimycotic. Cells were incubated at 37 °C in 5% CO_2_. H9c2 cells were cultured for 15 to 25 passages. The pcDNA3-GATA4-HA, pcDNA3.1-GATA6-V5-His-Topo, pGCN-SRF-HA, or pcDNA3-Nkx2.5 construct was transfected into H9c2 cells using Plus and Lipofectamine reagent, according to the manufacturer’s guidelines. After 48 h of transfection, cells were harvested and Nox2 mRNA levels were analyzed by qRT-PCR.

### Promoter assay

For the promoter assays, H9c2 cells were plated in 24-well plates and were transfected with pcDNA3-GATA4-HA or pcDNA3-Nkx2.5 in the presence of the pGL3‒Nox2 (‒541 bp) promoter luciferase construct and pCMV-β-galactosidase using Plus and Lipofectamine reagents (Invitrogen) according to the manufacturer’s instruction. After overnight serum starvation (0.5% FBS DMEM), cells were exposed to GA (100 µM) for 24 h. After cell harvesting and extraction using reporter lysis buffer (Promega, USA), luciferase activities were measured (Promega, USA) and normalized against β-galactosidase activity as an internal control.

### siRNA Transfection

For gene knockdown, H9c2 cells were transfected with negative control siRNA or rat GATA4 siRNA (100 nM, Santa Cruz) using RNAiMAX reagent (Invitrogen, Massachusetts, USA), according to the manufacturer’s guidelines. After transfection, cells were serum starved (0.5% FBS DMEM) overnight, exposed to angiotensin (Ang) II (1 µM, EMD Millipore, Billerica, MA, USA) for 24 h, harvested, and Nox activity was determined as mentioned under.

### Chromatin immunoprecipitation (ChIP) assays

ChIP assays were performed as described previously^31^. Briefly, H9c2 cells were transfected with pcDNA3-GATA4-HA or empty vector using Plus and Lipofectamine reagents (Invitrogen). After serum starvation, cells were treated with GA (100 µM) for 6 h. The concentration of GA did not cause cytotoxicity to the H9c2 cells. Cells were cross-linked with 1% formaldehyde for 10 min. The sonicated chromatin was immunoprecipitated with HA or normal mouse IgG antibody overnight and collected with protein A-agarose/salmon sperm DNA beads. Purified DNA was performed using a SYBR green PCR kit, amplifying the four GATA binding factor sites in the Nox2 promoter (−419 ~ to −19). ChIP PCR primers used are shown in Supplementary Table 1.

### Nitric oxide (NO) production assay

NO level was measured using Griess assay. To determine serum NO levels, serum was separated from blood samples by centrifugation (3000 rpm for 7 min at 4 °C) and was stored at −80 °C, until assayed. Hundred microliters of serum was added into a 96-well plate and 50 µl of 1% sulfanilamide in 5% phosphoric acid was added followed by a 10-min incubation in the dark. Fifty microliters of 0.1% N-1-napthylethylenediamine dihydrochloride in water was added to the reaction for 10 min and absorbance was measured at 570 nm.

For heart NO determination, 50–200 µg protein was diluted in distilled water and deproteinized using 5% trichloroacetic acid (TCA). After centrifugation (13000 rpm for 20 min), the supernatant was used for NO evaluation.

### NADPH oxidase (Nox) assay

Heart tissues were homogenized using lysis buffer (20 mM KH_2_PO_4_, 1 mM ethylene glycol tetraacetic acid (EGTA), pH 7.0, 1 mM DTT, 1 mM PMSF, 0.5 mM Na_3_VO_4_, 5 mM NaF, and protease inhibitor cocktail). To measure superoxide production, 100-µg protein lysate was diluted in assay buffer (50 mM KH_2_PO_4_, 1 mM EGTA, 150 mM sucrose, pH 7.0). NADPH (100 µM) and lucigenin (10 µM) was added to the protein lysates in 96-well plates. Luminescence was measured over 10 min (Centro XS^3^ LB960 microplate luminometer, Berthold Technologies, Bad Wildbad, Germany). Buffer blank was subtracted from each reading and data are expressed as relative light units produced per minute per milligram of protein. For Nox activity in H9c2 cells, cells were serum starved (0.5% FBS DMEM) overnight and then co-treated with Ang II (1 µM) and GA (100 µM) for 24 h. Nox activity was measured as indicated above for heart samples.

### Malondialdehyde (MDA) assay

As an indicator of lipid peroxidation, we measured the levels of malondialdehyde in rat heart tissue using thiobarbituric acid reactive substances (TBARS) according to the OxiSelect TBARS assay kit’s protocol (Cell Biolabs, Inc.). Heart tissue was homogenized with PBS containing butylated hydroxytoluene (BHT) on ice. After centrifugation at 10,000 g for 5 min, the supernatant was obtained. Lipid peroxidation assays were performed according to manufacturer’s protocol. MDA standards and samples were measured in duplicate. The level of MDA was determined spectrophotometrically using a UV-VIS spectrophotometer (Thermo Fisher Scientific, Finland) at 532 nm. MDA levels are expressed as nmol/mg of protein.

### Statistical analyses

Data are presented as the mean ± SE. Effects of rat strain and GA treatment on systolic blood pressure or body weight at different times (8, 12, and 24 weeks) were analyzed using a two-way ANOVA test. In these three groups, changes in systolic blood pressure or body weight according to time interval were analyzed using the Jonckheere-Terpstra trend test by SPSS version 22.0 software (SPSS, Chicago, IL, USA). All graphing was performed using GraphPad Prism version 5.03 software (GraphPad Software, San Diego, CA, USA), respectively.

## Results

### GA Reduces Blood Pressure, Vascular Remodeling, and Contraction in SHRs

To investigate whether GA regulates blood pressure in essential hypertension, we measured systolic blood pressure at 8, 12, and 24 weeks in SHRs. GA was administered to SHRs for 16 weeks. Eight-week-old SHRs showed a significantly elevated systolic blood pressure compared to WKY rats (165.6 ± 13.1 versus 123.4 ± 11.6 mm Hg; Fig. 1A). Twelve-week-old SHRs presented higher systolic blood pressure compared to control WKY rats (185.0 ± 12.2 versus 128.2 ± 10.4 mm Hg; Fig. 1A). GA-treated SHRs showed lower systolic blood pressure, compared to untreated SHRs, after four weeks of GA treatment (164.2 ± 2 versus 185.0 ± 12.2 mm Hg). Twenty-four-week-old SHRs showed the maximum systolic blood pressure compared to control WKY rats (200.1 ± 8.2 versus 128.5 ± 7.3 mm Hg; Fig. 1A). After 16 weeks of GA treatment, SHRs showed an even greater reduction in systolic blood pressure, compared with untreated SHRs (170.5 ± 15.8 versus 200.1 ± 8.2 mm Hg). In addition, we examined the changes in systolic blood pressure according to time point using the Jonckheere-Terpstra trend test. Changes in systolic blood pressure in WKY rats were not seen. However, a change in systolic blood pressure in SHRs was observed (p = 0.001).

To determine whether GA affects body weight, we measured body weights at the same time points at which the systolic blood pressure was measured. There was no significant difference in body weight between the groups at 8 weeks. At 12 weeks, GA-treated SHRs had a lower weight than control WKY rats. However, SHRs and GA-treated SHRs showed a significant reduction in average body weight compared to WKY rats, at 24 weeks (Fig. 1B). Furthermore, the average body weight of GA-treated SHRs was significantly lower than that of untreated SHRs (381.4 ± 17.4 versus 405.9 ± 14.1 g). In addition, the Jonckheere-Terpstra trend test showed increasing body weight of three different rats according to the age (p = 0.000).

Renin-angiotensin-aldosterone system (RAAS) is an important hormone system that regulates blood pressure^35^. We investigated angiotensin II type 1 receptor (AT1) and angiotensin converting enzyme (ACE1) mRNA levels in the aorta. Both AT1 and ACE1 mRNA levels were increased significantly in SHRs, compared to WKY rats, and this increase was completely abolished by GA treatment (Fig. 1C). Furthermore, GA treatment significantly reduced the increased transcript levels of AT1 in the heart and kidney cortex of SHRs (see Supplemental Fig. 1A,B). In addition, we observed similar results for AT1 and ACE1 protein levels in aortas (Fig. 1D). GA treatment significantly reduced aortic ACE1 and AT1 protein levels in SHRs (see Supplemental Fig. 2).

We further investigated how gallic acid affected the response to angiotensin II treatment in rat aortic rings. Vascular constriction was obtained following angiotensin II treatment in gallic acid- or vehicle-treated endothelium-intact aortic rings. The tension is expressed as a percentage in relation to initial contractions in response to 50 mM KCl. Pretreatment with gallic acid (1.0 mM or 3.0 mM) inhibited the vascular contractility in response to angiotensin II (see Supplemental Fig. 3). Next, we examined arterial remodeling using immunohistochemistry. As shown in Fig. 1E,F, the arterial wall was considerably thicker in SHRs, compared to WKY rats; the increased thickness was significantly reduced by GA treatment.

To further confirm the blood pressure-lowering effect of GA, we investigated vascular contractility in rat aortic rings and mesenteric arteries. GA treatment reduced U46619-induced vascular contractile response in both endothelium-intact and endothelium-denuded aortas (Fig. 1G). However, there was no difference between the responses in endothelium-intact and endothelium-denuded aortas, at each GA concentration tested (ED_50_ (−log M) 2.96 ± 0.05 versus 2.91 ± 0.01, respectively; Supplementary Table 2). These results indicate that GA acts mainly on vascular smooth muscle cells, but not endothelial cells. Similar results were observed in mesenteric arteries. In the mesenteric arteries, the GA cumulative curve resulted in a similar relaxation response in the U46619 treatment (ED_50_ (−log M) 3.50 ± 0.06, Supplementary Table 2). To further identify whether GA relaxes vascular contraction through activation of the nitric oxide (NO) pathway, we pretreated endothelium-intact aortic rings with N^G^-nitro-_L_-arginine methyl ester (_L_-NAME, 1.0, 10, or 100 µM) or vehicle for 30 min. GA was added cumulatively (0.01–3.0 mM) to induce relaxation when vascular contraction induced by U46619 (30 nM) reached a plateau. As shown in Supplemental Fig. 4, _L_-NAME did not block the vascular relaxation induced by GA in endothelium-intact aortic rings. The ED_50_ values were similar between L-NAME treatment groups (Supplementary Table 3).

### GA Attenuates Left Ventricular Hypertrophy in SHRs

**Figure 2:**
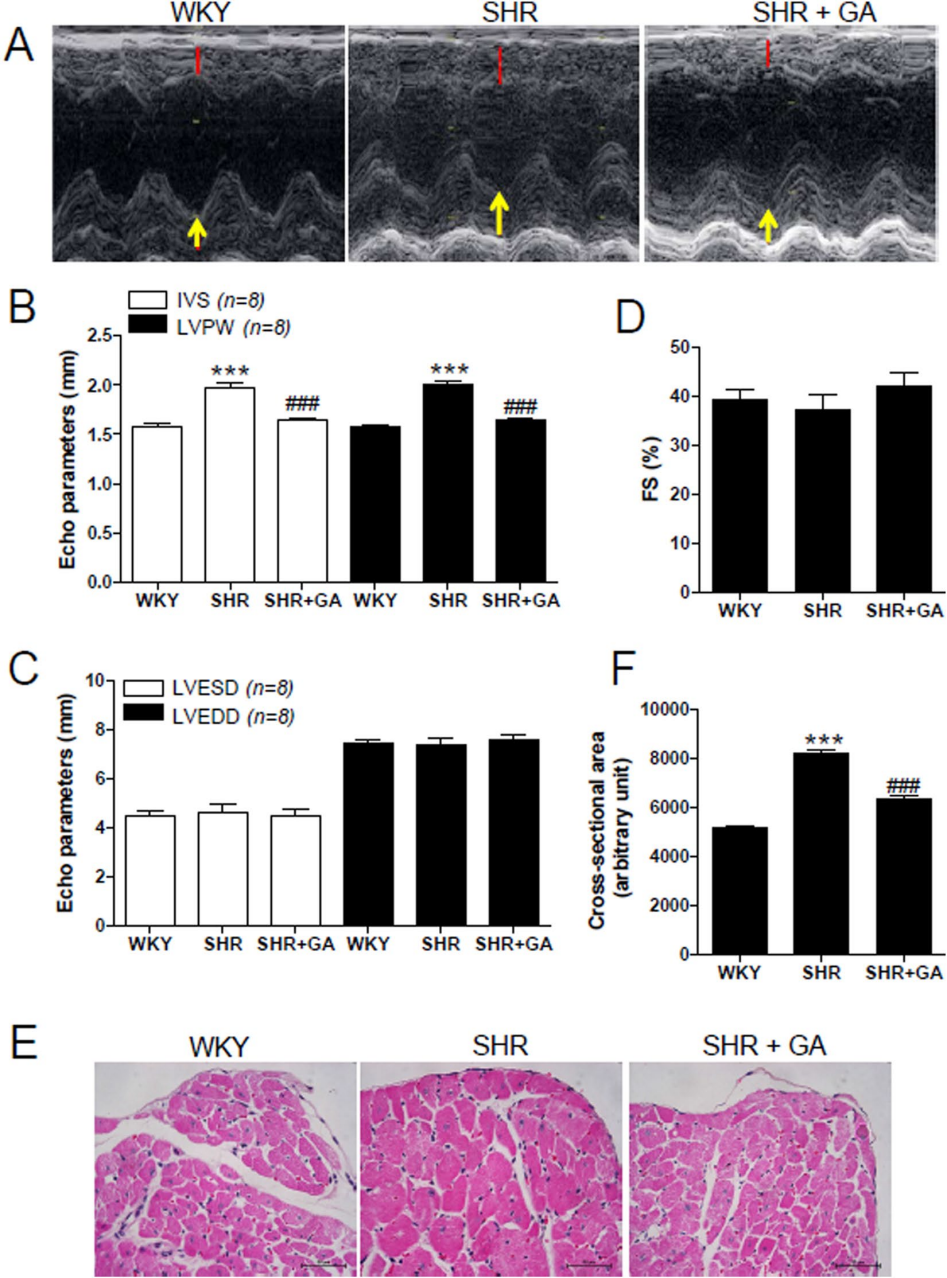
GA attenuates left ventricular hypertrophy in SHRs. (**A**) Representative M-mode echocardiogram of 24-week-old rats from the three groups. Red lines and yellow arrows indicate left ventricular septum and posterior wall thickness, respectively. (**B**–**D**) Echocardiographic parameters, including IVS, LVPW, LVESD, LVEDD, and FS were evaluated in rats from WKY, SHR, and SHR+GA groups (n = 8 per group). ***P < 0.001 versus WKY at 24 weeks. ^##^ *P* < 0.01 and ^###^ *P* < 0.001 versus SHR at 24 weeks. (**E**,**H** and **E**) stain (scale bar, 50 µm) was performed to evaluate myocyte size. (**F**) Cross-sectional areas of left ventricle were evaluated (n = 8 per group). ***P < 0.001 versus WKY at 24 weeks. ^###^ *P* < 0.001 versus SHR at 24 weeks.

SHRs manifest left ventricular hypertrophy^36^. We found that the total heart weight or left ventricular weight of SHRs was significantly greater than that of WKY rats (see Supplemental Fig. 5A,B). GA treatment in SHRs reduced the total heart weight to tibia length ratio (mg/mm) or left ventricular (LV) weight to tibia length ratio (mg/mm). To further evaluate whether GA affects heart function and wall thickness in SHRs, we performed echocardiography. Figure 2A shows representative M-mode images. Interventricular septum (IVS) and left ventricular posterior wall (LVPW) thickness were significantly increased in 24-week-old SHRs, compared to that in WKY rats. The increased IVS and LVPW thickness were significantly reduced by GA administration (Fig. 2B). The end-systolic and end-diastolic dimensions of LV were not different among the three groups (Fig. 2C). In addition, fractional shortening (FS) was comparable among the three groups (Fig. 2D). To determine whether GA exerts its anti-hypertrophic effect at cellular levels, we performed H&E staining to measure the cross-sectional myocyte size. The cross-sectional area was greater in SHRs, compared to that in WKY rats, and this increase was attenuated by GA treatment (Fig. 2E,F).

### GA Suppresses Expression of Cardiac-Specific Transcription Factors in SHRs and Angiotensin II-Treated H9c2 Cells

**Figure 3:**
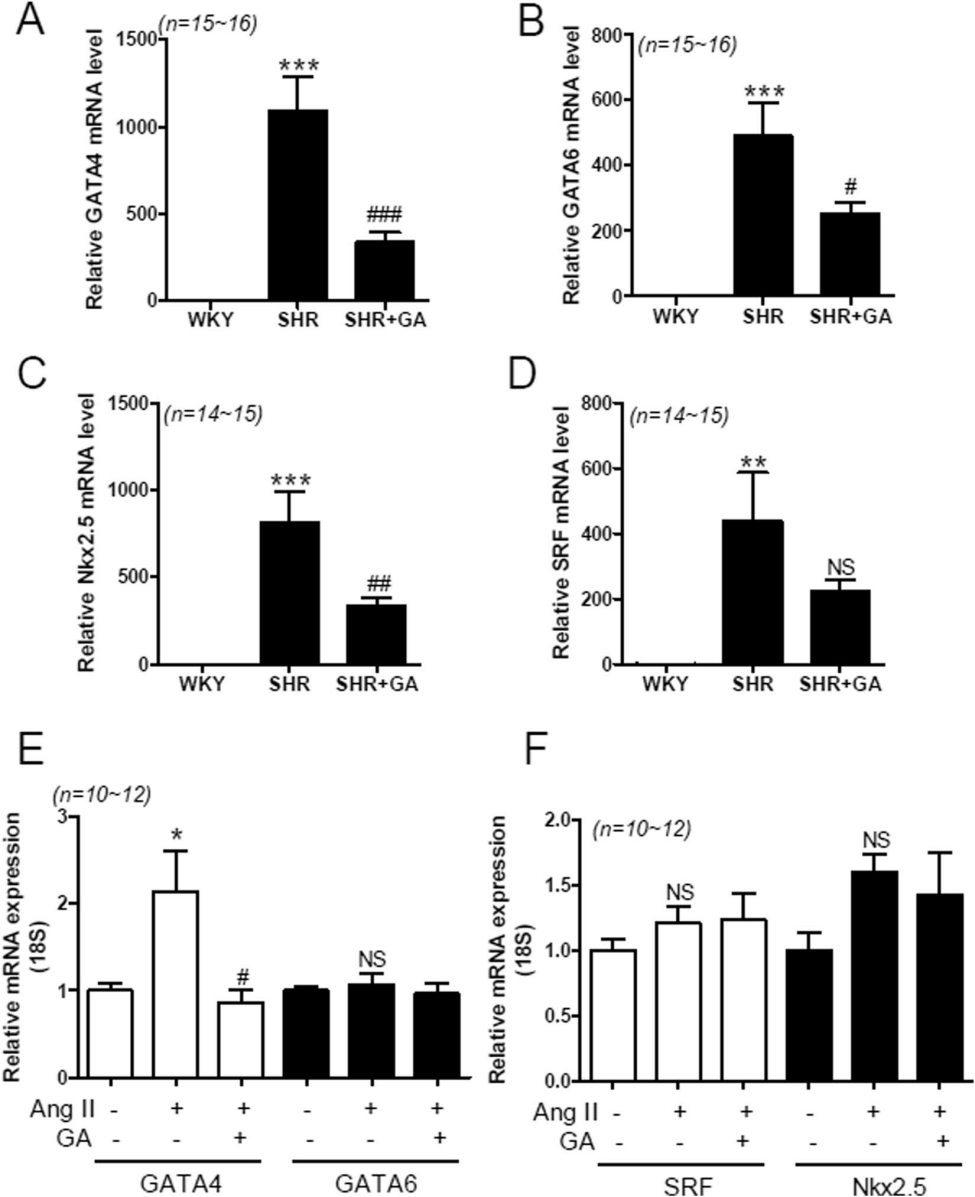
GA reduces expression of cardiac-specific transcription factors in SHRs and angiotensin II-treated H9c2 cells. (**A**–**D**) Transcript levels of GATA4, GATA6, Nkx2-5, and SRF were evaluated by RT-PCR, for the three groups, at 24 weeks (n = 14~16 per group). The transcript levels have been normalized to 18S and presented as a relative value. **P < 0.01 and ***P < 0.001 versus WKY. ^#^ *P* < 0.05, ^##^ *P* < 0.01, and ^###^ *P* < 0.001 versus SHR. NS, not significant. **(E**,**F**), H9c2 cells were treated with either vehicle or GA (100 µM) for 9 h after exposure to angiotensin II (1 µM). The values are the mean ± SE of at least three independent experiments. (**E**) GATA4 and GATA6 mRNA were quantified using qRT-PCR. *P < 0.05 versus vehicle-treated cells. ^#^ *P* < 0.05 versus angiotensin II-treated cells. NS, not significant. (**F**) Nkx2-5 and SRF transcript levels in H9c2 cells. NS, not significant.

Cardiac enlargement can be regulated by cardiac-specific transcription factors^37^. To investigate the expression of cardiac-specific transcription factors, we performed quantitative RT-PCR using rat heart samples from the three groups: WKY, SHRs, and SHRs plus GA. We found that GATA4 and GATA6 mRNA levels were upregulated in SHRs, compared to WKY rats, and this upregulation was reduced by GA administration (Fig. 3A,B). We examined two more cardiac-specific transcription factors, Nkx2-5 and SRF. GA treatment significantly decreased the Nkx2-5 mRNA levels in SHR hearts (Fig. 3C). However, the reduction in the SRF transcript level by GA was not statistically significant (Fig. 3D). It has been reported to increase angiotensin II concentration in the blood plasma of SHRs^38,39^. We sought to determine whether the increased transcription factor levels in SHR hearts could be observed in angiotensin II-treated H9c2 cells. We observed similar results in H9c2 cells. The GATA4 transcript was upregulated in response to angiotensin II treatment and this increase was abolished by GA treatment (Fig. 3E). However, GATA6, SRF, and Nkx2-5 upregulation was not seen in angiotensin II-treated H9c2 cells (Fig. 3F).

### GA Attenuates Nox2 Expression in SHRs and Angiotensin II-Treated H9c2 Cells

**Figure 4:**
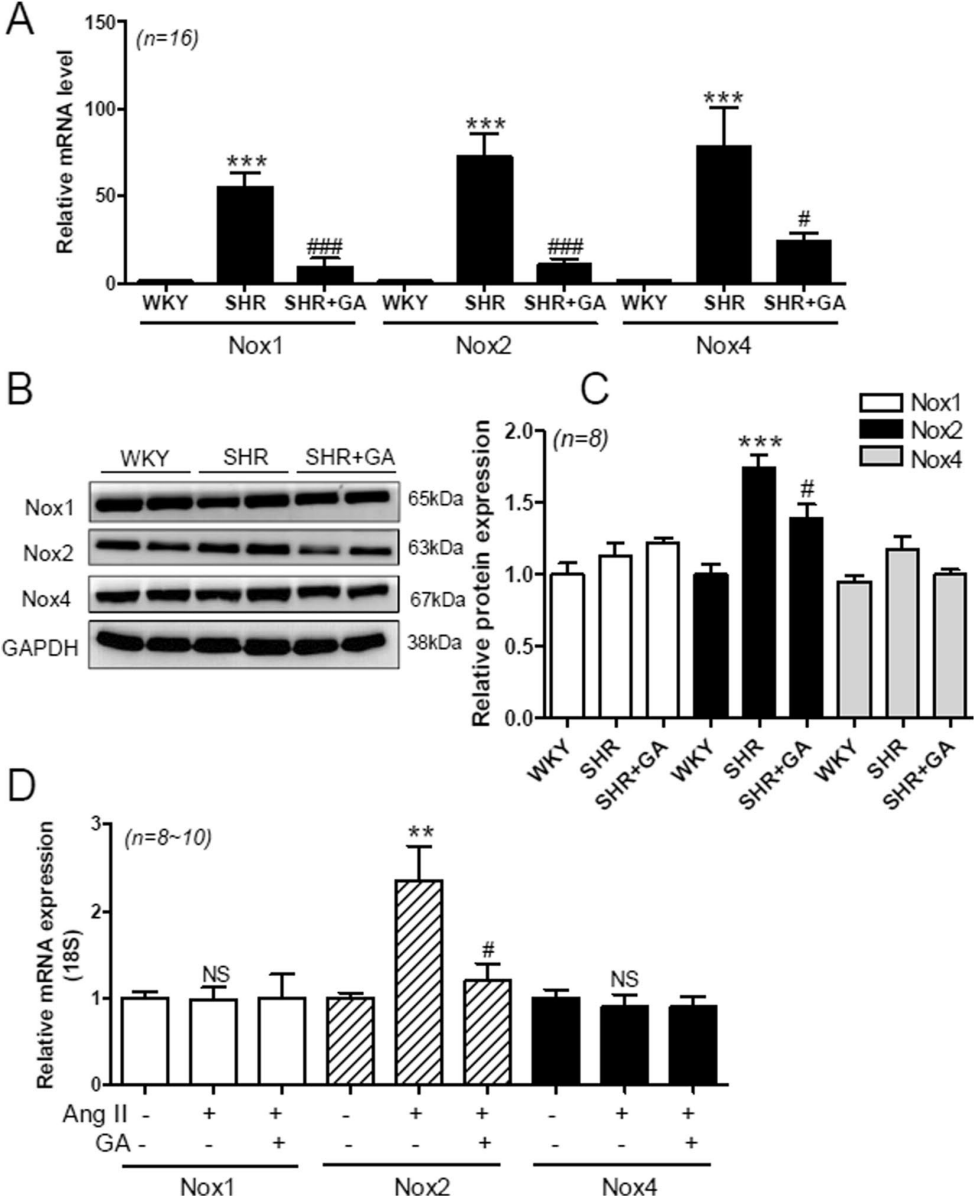
GA attenuates Nox2 expression in SHRs and angiotensin II-treated H9c2 cells. (**A**) The mRNA levels of Nox1, Nox2, and Nox4 were analyzed using qRT-PCR. The transcript levels have been normalized to 18 S and presented as a relative value. ***P < 0.001 versus WKY. ^#^ *P* < 0.05 and ^###^ *P* < 0.001 versus SHR. (**B**) Representative immunoblots. Nox1, Nox2, and Nox4 protein expression in rat hearts from the WKY, SHR, and SHR+GA groups, was determined using western blot analysis. GAPDH was used as the loading control. (**C**) The protein expression of Nox1, Nox2, and Nox4 was quantified by densitometry. ***P < 0.001 versus WKY. ^#^ *P* < 0.05 versus SHR. (**D**) The mRNA levels of Nox1, Nox2, and Nox4 were determined using H9c2 cells treated with the vehicle (control), with vehicle + Ang II (1 µM), or with Ang II (1 µM) + GA (100 µM) for 9 h. The values are the mean ± SE of at least three independent experiments. **P < 0.01 versus vehicle-treated cells. ^#^ *P* < 0.05 versus angiotensin II-treated cells. NS, not significant.

NADPH oxidase (Nox) produces reactive oxygen species (ROS), which are implicated in hypertension and cardiac hypertrophy^40^. Therefore, we examined whether Nox gene expression is upregulated in SHR hearts. We found that cardiac Nox1, Nox2, and Nox4 mRNA levels were significantly increased in SHRs, compared to WKY rats. This upregulation was decreased by GA treatment (Fig. 4A). Next, we investigated Nox protein expression. As shown in Fig. 4B, Nox2 protein levels were increased in SHRs, compared to WKY rats. The increased Nox2 protein expression was significantly attenuated by GA treatment (Fig. 4B,C). However, there was no significant difference in Nox1 and Nox4 protein expression among the three groups, even though Nox4 protein levels showed a tendency to increase (Fig. 4B,C).

We then sought to determine whether the Nox upregulation seen in SHRs occurs in H9c2 cells treated with angiotensin II. Nox2 mRNA levels were upregulated in response to angiotensin II treatment (Fig. 4D). This upregulation was significantly abolished by GA treatment. However, Nox1 and Nox4 mRNA expression was unchanged after angiotensin II or GA treatment.

### GA Attenuates GATA4-Mediated Nox2 Promoter Activation in H9c2 Cells

**Figure 5:**
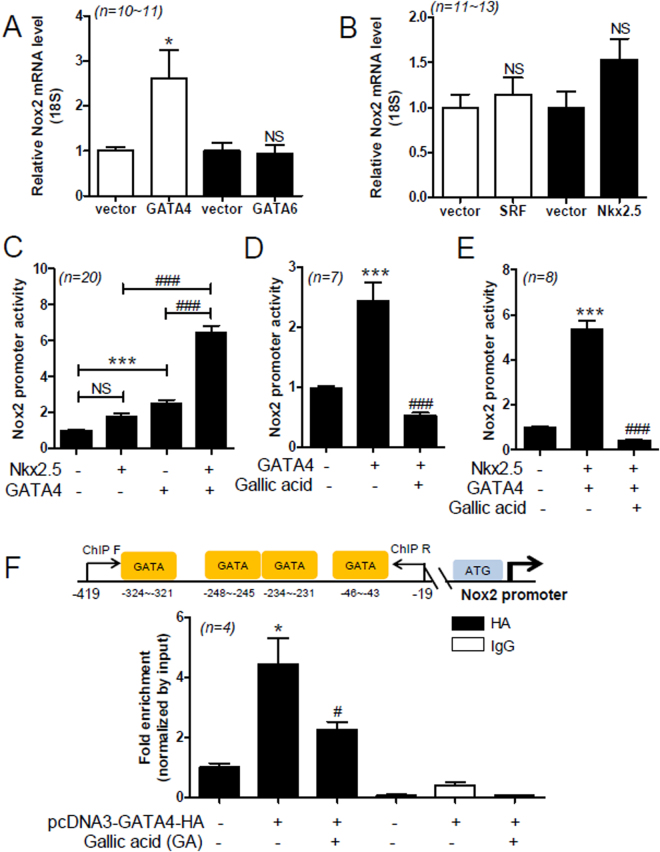
GA attenuates GATA4/Nkx2-5-mediated Nox2 promoter activation in H9c2 cells. (**A,B**) The pcDNA3-GATA4-HA, pcDNA3.1-GATA6-V5-His-TOPO, pGCN-SRF-HA, or pcDNA3-Nkx2-5 constructs were transfected into H9c2 cells for 2 days. Nox2 mRNA levels were evaluated by quantitative RT-PCR. Data are presented as the mean ± SE with at least 4 independent experiments. *P < 0.05 versus empty vector. NS, not significant. (**C**) H9c2 cells were co-transfected with Nox2 promoter luciferase, β-galactosidase expression vector, empty vector, pcDNA3-GATA4-HA, or pcDNA3-Nkx2-5 construct. Nox2 promoter assay was performed. The values are the mean ± SE of at least five independent experiments. ***P < 0.01 versus empty vector. ^###^ *P* < 0.001 versus pcDNA3-GATA4-HA or pcDNA3-Nkx2-5 transfection. Data are presented as the mean ± SE with at least 5 independent experiments. (**D**‒**E**) H9c2 cells were transfected with pcDNA3-GATA4-HA and pcDNA3-Nkx2-5 and then incubated with GA (100 µM) for 24 h. The values are the mean ± SE of at least four independent experiments. ***P < 0.001 versus empty vector. ^###^ *P* < 0.001 versus GA-treated group. Data are presented as the mean ± SE with at least 3 independent experiments. (**F**) For the ChIP assays, pcDNA3-GATA4-HA or empty vector was transfected into H9c2 cells. After treatment with GA (100 µM) for 6 h, chromatin DNA complexes were immunoprecipitated by anti-HA antibody or normal mouse IgG. GATA indicates GATA binding site. ChIP Forward (**F**) and ChIP Reverse (**R**) indicate the PCR primer sets (−419 to −19). Schematic diagram shows the candidate GATA binding sites in the rat Nox2 promoter. Data are presented as the mean ± SE with at least 3 independent experiments.

To determine whether the expression of cardiac-specific transcription factors is related to the regulation of Nox2 in hypertension, H9c2 cells were transfected with transcription factors, including GATA4, GATA6, Nkx2-5, or SRF. Forced transfection with GATA4, GATA6, SRF, or Nkx2-5 significantly increased their exogenous mRNA levels in H9c2 cells (see Supplemental Fig. 6). Forced expression of GATA4 significantly increased Nox2 mRNA levels, while GATA6 and SRF expression failed to induce it (Fig. 5A,B). However, Nkx2-5 seemed to increase Nox2 mRNA levels (Fig. 5B).

To determine whether Nox2 upregulation induced by transcription factors is regulated at the promoter level, we performed Nox2 promoter assay. Nox2 promoter has GATA-binding sequences. Transfection with GATA4 significantly increased the activity of the Nox2 promoter (see Supplemental Fig. 7A), while Nkx2-5 did not significantly increase it (see Supplemental Fig. 7B). However, GATA6 and SRF did not cause any increase in the Nox2 promoter activity (see Supplemental Fig. 7C,D). Therefore, we sought to determine the synergistic effect of GATA4 and Nkx2-5 on Nox2 promoter activity. Transfection with GATA4 increased Nox2 promoter activity about 2.5-fold, compared to transfection with the vector alone (Fig. 5C). Co-transfection with Nkx2-5 and GATA4 further increased the activity of the Nox2 promoter, compared to transfection with GATA4 or Nkx2-5 alone (Fig. 5C, fourth bar). GA abolished the GATA4-mediated increase in Nox2 promoter activity (Fig. 5D). Furthermore, it also suppressed the synergistic effect of GATA4 and Nkx2-5 on Nox2 promoter activity (Fig. 5E). To investigate the regulatory mechanism by which GA downregulates Nox2 expression, we performed chromatin immunoprecipitation (ChIP) assay. Rat Nox2 promoter has several GATA-binding sites. We used primers including four GATA-binding sites. The ChIP assay results suggest that GA treatment decreases the DNA-binding activity of GATA4 in the rat Nox2 promoter (Fig. 5F).

### GA Recovers NO Production and Reduces MDA and Nox Activity *in vivo* and *in vitro*

**Figure 6:**
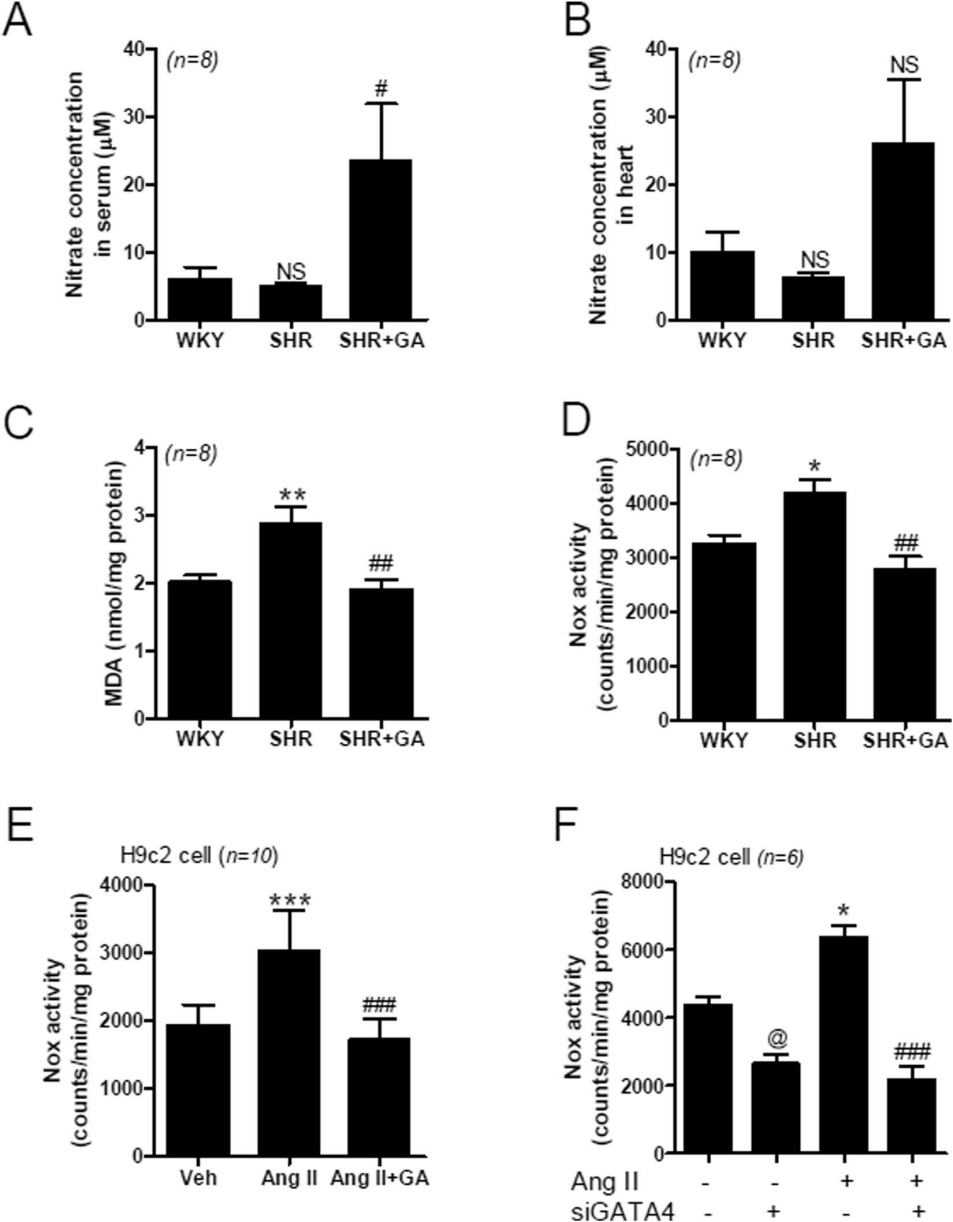
GA regulates NO production and Nox activity *in vivo* and *in vitro*. (**A**) Nitric oxide (NO) production in serum was evaluated using Griess reagent. Data are expressed as means ± SE (n = 8 rats per group). NS, not significant. ^#^ *P* < 0.05 versus SHR. (**B**) NO production in heart tissues from WKY, SHR, and SHR+GA groups, was determined. Data are expressed as means ± SE (n = 8 rats per group). (**C**) Malondiadehyde (MDA) levels in the heart of WKY, SHR, and SHR+GA. Data are presented as the mean ± SE (n = 8 rats per group). **P < 0.01 versus WKY. ^##^ *P* < 0.01 versus SHR. (**D**) Nox activity in rat hearts from the WKY, SHR, and SHR+GA groups was measured by lucigenin chemiluminescence assay. Data are expressed as means ± SE (n = 8 rats per group). *P < 0.05 versus WKY. ^##^ *P* < 0.01 versus SHR. (**E**) Nox activity was measured in H9c2 cells treated with angiotensin II (Ang II, 1 µM) or GA (100 µM) for 24 h. The values are the mean ± SE of at least four independent experiments. ***P < 0.001 versus vehicle group. ^###^ *P* < 0.001 versus Ang II-treated group. (**F**) After GATA4 siRNA transfection, H9c2 cells were incubated with Ang II (1 µM) to determine Nox activity. The values are the mean ± SE of at least three independent experiments. ^@^ *P* < 0.05 versus vehicle-treated siControl group. *P < 0.05 versus vehicle-treated siControl group. ^###^ *P* < 0.001 versus Ang II-treated siControl group.

To determine oxidative stress in hypertension, we measured NO levels in the serum and heart, using Griess reagent. The serum levels of NO in WKY rats and SHRs were not different, but were increased significantly after GA treatment (Fig. 6A). Although the NO levels in the heart showed a similar pattern, the increase was not statistically significant (Fig. 6B). In addition, we measured the content of malondialdehyde (MDA) in rat heart tissue. The MDA levels were significantly increased in SHRs compared to in the sham. The increase was reduced by GA treatment (Fig. 6C). To determine superoxide production in the heart tissues and H9c2 cells, we measured Nox activity using lucigenin-enhanced chemiluminescence. The Nox activity was significantly higher in SHR hearts, compared to that in WKY rat hearts; this increase was abolished by GA treatment (Fig. 6D). GA also suppressed angiotensin II-induced Nox activity in H9c2 cells (Fig. 6E). To further investigate whether GATA4 can mediate angiotensin II-induced Nox activity, we knocked down GATA4 and measured Nox activity in response to angiotensin II. Transfection with GATA4 siRNA successfully reduced the endogenous GATA4 mRNA level (see Supplemental Fig. 8). Nox activity was increased by angiotensin II treatment, and this increase was abolished by transfection with GATA4 siRNA (Fig. 6F).

## Discussion

GA has been shown to prevent streptozotocin-induced hyperlipidemia, hypertension, and LVH^22^. However, the mechanism behind the beneficial effects of GA remains unknown. In this study, we demonstrate that long-term administration of GA reduces hypertension, LVH, and oxidative stress in animal model of essential hypertension.

The regulatory mechanism by which GA affects blood pressure in the vasculature may be explained in several ways. First, GA regulates the components of RAAS. AT1 mRNA levels are downregulated by GA treatment in the aorta, heart, and kidney cortex of SHRs. Similarly, GA reduced the enhanced ACE1 mRNA levels in SHR aortas. In addition, GA administration resulted in the reduction of AT1 and ACE1 protein expression in SHR aortas. This is consistent with a previous study reporting GA-mediated inhibition of ACE1 and anti-hypertensive effect in SHRs^41^. Second, GA exerts relaxation of vascular contraction in both a large conduit vessel (aorta) and small resistance vessels (mesenteric arteries). Third, GA-mediated weight loss may be implicated in the reduction of blood pressure. Weight reduction leads to lowered blood pressure. Our findings warrant further research; however, a recent study has demonstrated that an angiotensin II receptor antagonist (telmisartan) induces weight loss via the angiotensin-(1–7)/Mas-dependent pathway^42^. Fourth, GA reduces the increase in aortic wall thickness induced in SHRs.

We observed beneficial effects of GA in essential hypertension. GA ameliorated LVH, as determined by echocardiography. In addition, by measuring the cross-sectional area, we found that GA treatment significantly diminished enlarged cardiomyocyte size. The anti-hypertrophic effect of GA in SHRs is supported by our previous paper, indicating that GA prevents β-adrenergic agonist-induced cardiac hypertrophy^31^. Another paper presented similar results showing that GA decreased the increased heart weight to body weight ratio on streptozotocin-induced diabetic rats^22^. Cardiac-specific transcription factors are closely implicated in cardiac hypertrophy^43^. In this study, we found that GA decreased the increased mRNA levels of GATA4, GATA6, SRF, and Nkx2-5 induced in SHRs. The GATA4 mRNA level was also induced in response to angiotensin II treatment in H9c2 cells. These findings imply that the reduction of cardiac hypertrophy by GA is associated with GATA4 downregulation. Indeed, GATA4 overexpression induces cardiac hypertrophy *in vivo* and *in vitro* ^44^, suggesting GATA4 is an important hypertrophic regulator.

Oxidative stress plays an important role in hypertension. Notably, MDA level, which is a marker of oxidative stress, was increased in the hearts of SHRs compared to those of control rodents. Treatment with GA reduced MDA levels in heart tissue. NADPH oxidase (Nox) is related to reactive oxidase stress (ROS) in activated RAAS. Furthermore, Nox activity was increased in SHR hearts and angiotensin II-treated H9c2 cells and was reduced by GA treatment. Similarly, Nox activity was increased in SHR aortas compared with those in WKY rats^45,46^. The cardiac transcription factor GATA4 was involved in the reduction of Nox activity by GA treatment.

Nox1 and Nox4 are expressed in vascular smooth muscle cells, whereas Nox2 and Nox4 are highly expressed in endothelial cells^47^. Similar to previous reports, our results show enhanced Nox1, Nox2, and Nox4 mRNA levels in SHR hearts^48–50^. Chabrashvili T *et al*. reported that p47phox mRNA levels are higher in SHR kidneys than in WKY rats^51^. Nox4 mRNA expression has been shown to be higher in the basilar artery in SHRs^52^. The differences between our results and those of previous studies appear to have been caused by the use of different organs (heart, kidney, and artery) and animals of varying ages for experiments.

In the present study, GA treatment reduced cardiac Nox1, Nox2, and Nox4 transcript levels, and attenuated cardiac Nox2 protein expression in SHRs. However, we are unable to explain the discrepancy observed between Nox mRNA and protein expression. We found that angiotensin II increases the mRNA levels of Nox2, but not Nox1 or Nox4, in H9c2 cells. These results suggest that Nox2 plays a more important role than the other Noxs in mediating oxidative stress response in cardiomyocytes. Nox2 is activated by G-protein-coupled receptor agonists, such as angiotensin II or endothelin-1^53^, as well as aortic banding in pigs^54^. Relevant to Nox2 in cardiomyocytes, Nox2 expression was increased in human cardiomyocytes after acute myocardial infarction^55^. In the present study, essential hypertension induces cardiac hypertrophy, which is closely involved in upregulation of Nox2.

Therefore, we focused on the role of Nox2 in hypertension. Transcriptional regulation of Nox1 is mediated by the direct binding of GATA-binding factor to the Nox1 promoter^56^. Here, we investigated whether cardiac Nox2 expression is regulated by cardiac-specific transcription factors, including GATA4, GATA6, SRF, and Nkx2-5. Interestingly, Nox2 upregulation was induced by the synergistic action of GATA4 and Nkx2-5. A possible mechanism is that Nkx2-5 overexpression increased the GATA4 mRNA levels in H9c2 cells in a dose-dependent manner (see Supplemental Fig. 9). Consistent with our results, an adenovirus expressing Nkx2-5 has been shown to increase GATA4 promoter activity *in vitro* ^57^. Even though we have not demonstrated a direct association between Nkx2-5 and GATA4, there is some evidence to suggest that Nkx2-5 physically interacts with GATA4^58^. A potential regulatory mechanism by which GA regulates Nox2 expression is that it interferes with the binding of GATA4 to the Nox2 promoter DNA.

A limitation of the current study is that the GA concentration used in the cell cultures is not precisely correlated with chronic doses observed *in vivo*. Despite long-term oral administration of GA to SHRs, systolic blood pressure was not restored to that of control WKY rats. Thus, other mechanisms may be involved in the reduction of blood pressure by GA treatment of SHRs. GA was reported to have diuretic activity in Wistar albino rats^59^. Even though we observed body weight loss in GA-treated SHRs compared to SHRs, we did not examine urine volume in the present study. Therefore, we cannot rule out the possibility of a diuretic action of GA in the regulation of blood pressure.

A pharmacological inhibitor (KN-93) of the calcium/calmodulin-dependent kinase (CaMKII)^60^ and transgenic expression of CaMKII peptide inhibitor in VSMC have been reported to attenuate hypertension after angiotensin II infusion by controlling baroreceptor function^61^. Moreover, CaMKII delta expression was increased in SHRs with cardiac hypertrophy^62^. We have evidence that GA downregulates CaMKII expression in SHRs (accepted article, Journal of Cellular and Molecular Medicine). Therefore, we speculate that another mechanism exists for the regulation of blood pressure by GA. We need to investigate the relevance of CaMKII to voltage-gated Ca^2+^ channels that control vascular tone.

Sympathetic nervous system activation increases and maintains blood pressure^7^. Epigallocatechin-3-O-gallate (EGCG), an ester of epigallocatechin and gallic acid, attenuates hypertension and circulating norepinephrine levels induced by SHRs^63^. Therefore, we cannot eliminate the relevance of GA to the regulation of the sympathetic nervous system in hypertension. Hypertension is a very complex disease and its pathophysiological mechanisms are diverse. Therefore, we did not investigate this in detail in the current study, as pointed out above.

In summary, we have demonstrated that GA lowers systolic blood pressure and LVH in rats with essential hypertension, and that it inhibits cardiac Nox2 expression and the Nox2-induced oxidative stress response through the knockdown of GATA4 or by interfering with the DNA-binding activity of GATA4. We suggest that GA is a novel promising therapy for the treatment of cardiovascular diseases, including hypertension and cardiac hypertrophy.

## Electronic supplementary material

Supplementary figures and information
