# Supplementary material for: Gallic Acid Reduces Blood Pressure and Attenuates Oxidative Stress and Cardiac Hypertrophy in Spontaneously Hypertensive Rats
Source: Sci Rep. 2017 Nov 15;7:15607. doi: 10.1038/s41598-017-15925-1 (PMC5688141; doi:10.1038/s41598-017-15925-1)

Gallic Acid Reduces Blood Pressure and Attenuates Oxidative Stress and Cardiac Hypertrophy in Spontaneously Hypertensive Rats

Li Jin[1,2+], Zhe Hao Piao[3+], Simei Sun[1], Bin Liu[3], Gwi Ran Kim[1], Young Mi Seok[4], Ming Quan Lin[5], Yuhee Ryu[1], Sin Young Choi[1], Hae Jin Kee[1,*], and Myung Ho Jeong[1,*]

1. Supplementary Table 1. Primers for reverse transcription-polymerase chain reaction (RT-PCR) and ChIP

Gene	Primer sequence (5′ to 3′)
18S rRNA (rat)	F: CATTCGAACGTCTGCCCTAT<br>R: GCCTTCCTTGGATGTGGTAG
AT1 (rat)	F: GGAAACAGCTTGGTGGTGAT<br>R: GGCCGAAGCGATCTTACATA
ACE1 (rat)	F: GTACAGAAGGGCTGGAATGC<br>R: CGTGCACTGCTTAATCCTGA
GATA4 (rat)	F: ACCAAAGCCTGCCTATGGCC<br>R: CCGCCAGGGACCCAGTAGTC
GATA6 (rat)	F: TGAACGGGACGTACCACCACCACC<br>R: ACAGTTCACGCACTCGCGGCTCTC
SRF (rat)	F: CCGCGTGAAGATCAAGATGGAGTT<br>R: TGCCAGGTAGTTGGTGATGGGGAA
Nkx2-5 (rat)	F: ACACACACGCCCTTCTCAGTCAAA<br>R: AAAATGTAGGGGCGGTTGGGAAAG
Nox1 (rat)	F: AGCCATTGGATCACAACCTC<br>R: TGGATGGGATTTAGCCAAGA
Nox2 (rat)	F: TGTCATTCTGGTGTGGTTGG<br>R: GAACCCCTGAGGAAGGAGAG
Nox4 (rat)	F: CTGGAAGAACCCAAGTTCCA<br>R: ACTGGCCAGGTCTTGCTTTA
ChIP (rat)	F: TATACATTACCCAGTGAGCACCAA<br>R: GGGGCAATATGTTTCCTTTTCTAT

F, forward; R, reverse.

Supplementary Table 2. ED_{50} values for gallic acid on vascular contraction induced by U46619 stimulus in rat aorta and mesenteric artery

	Aorta (endothelium intact) ED_{50} (-log M)	Aorta (endothelium denuded) ED_{50} (-log M)	Mesenteric artery (endothelium intact) ED_{50} (-log M)
U46619	2.96±0.05	2.91±0.01	3.50±0.06

Data are the mean ± SEM of four experiments.

Supplementary Table 3. ED_{50} values for vascular contraction response of endothelium-intact rat aortic rings to U46619 after L-NAME

Treatment (L-NAME, μM)	ED_{50} (-log M)
0	3.06±0.02
1.0	3.18±0.02
10	3.15±0.03
100	2.99±0.01

Data are the mean ± SEM of four experiments.

2. Supplemental Figures

Figure S1. GA decreases AT1 mRNA expression in heart and kidneys of SHRs. A-B, GA was administered to SHRs for 16 weeks. Total RNA was isolated from heart and kidney cortex tissues and AT1 mRNA expression was determined by qRT-PCR. The mRNA levels were normalized to 18S or GAPDH. ****P*<0.001 versus WKY. $^{\#}$*P*<0.05 versus SHR. $^{\#\#}$*P*<0.01 versus SHR.

Figure S2. GA decreases ACE and AT1 protein levels in aortic SHRs. A-B, GA was administered to SHRs for 16 weeks. ACE1 and AT1 protein levels were quantified using densitometry. **P*<0.05 and ****P*<0.001 versus WKY. $^{\#\#\#}$*P*<0.05 versus SHR.

Figure S3. GA decreases angiotensin II-induced vasoconstriction in rat aortic rings.

Angiotensin II (Ang II, 1.0 μM) was added to elicit tension 30 min after pretreatment with GA (0.3, 1.0, or 3.0 mM) or vehicle in intact aortic rings. Developed tension is expressed as a percentage of the maximal contraction to 50 mM KCl. Data are expressed as the means of five experiments, with vertical bars showing standard error of the mean. **P*<0.05 and ** *P*<0.01 as compared with Ang II.

Figure S4. L-NAME did not block vasorelaxation induced by GA in rat aortic rings.

Aortic rings were pretreated with N^{G}-nitro-L-arginine methyl ester (L-NAME, 1.0, 10, or 100 μM) or vehicle (0.1% dimethylsulfoxide) for 30 min. Gallic acid was added cumulatively to elicit relaxation when vascular contractions induced by U46619 (30 nM) reached a plateau in endothelium-intact rat aortic rings (n=4 per group). Relaxation is expressed as a percentage of

the maximal contraction. Data are expressed as the mean ± standard error of the mean.

Figure S5. GA attenuates left ventricular hypertrophy in SHRs. The ratios of total heart weight to tibia length and left ventricular heart weight/tibia length in WKY, SHR, SHR groups treated with GA for 16 weeks (n=8 per group). ***P<0.001 versus WKY. $^{\#}P$<0.05 versus SHR.

Figure S6. Cardiac transcription factors in H9c2 cells.

A-D, H9c2 cells were transfected with empty vector, pcDNA3-GATA4-HA, pcDNA3.1-GATA6-V5-His-Topo, pGCN-SRF-HA, or pcDNA3-Nkx2-5. Total RNA was isolated from cells and mRNA expression was determined by qRT-PCR. The relative expression levels were normalized to 18S. **P<0.01 versus WKY. ***P<0.001 versus WKY.

Figure S7. GATA4 increases Nox2 promoter activity in H9c2 cells.

A-D, H9c2 cells were transfected with -540 rat Nox2 luciferase construct and β-galactosidase expression vector along with either empty vector, pcDNA3-GATA4-HA, pcDNA3.1-GATA6-V5-His-Topo, pGCN-SRF-HA, or pcDNA3-Nkx2-5. ***P<0.001 versus empty vector. NS, not significant.

Figure S8. Knockdown of GATA4 decreases endogenous GATA4 mRNA levels in H9c2 cells.

H9c2 cells were transfected with either sicontrol or GATA4 siRNA for 48 h. Total RNA was isolated. GATA4 mRNA levels were determined by qRT-PCR. ***P<0.001 versus sicontrol.

Figure S9. Nkx2-5 augments GATA4 mRNA levels in H9c2 cells.

A-B, H9c2 cells were transfected with empty vector or pcDNA3-Nkx2-5 for 48 h. Nkx2-5 and GATA4 mRNA levels were determined by qRT-PCR. ***P<0.001 versus empty vector.

Figure S1

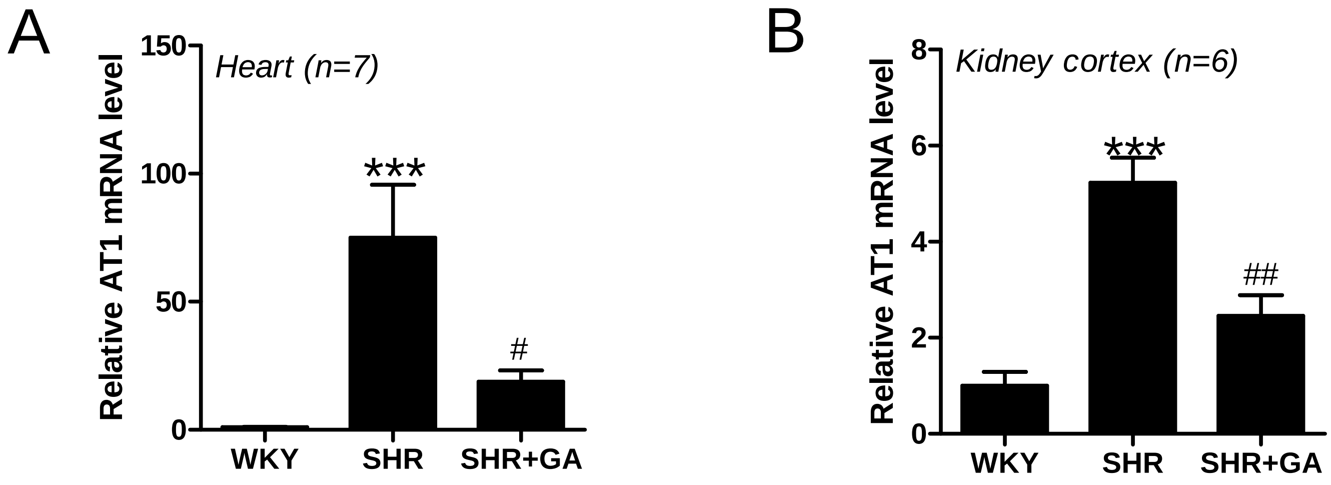
A
Heart (n=7)
Relative AT1 mRNA level
150
100
50
0
***
#
WKY
SHR
SHR+GA
B
Kidney cortex (n=6)
Relative AT1 mRNA level
8
6
4
2
0
***
##
WKY
SHR
SHR+GA

Figure S2

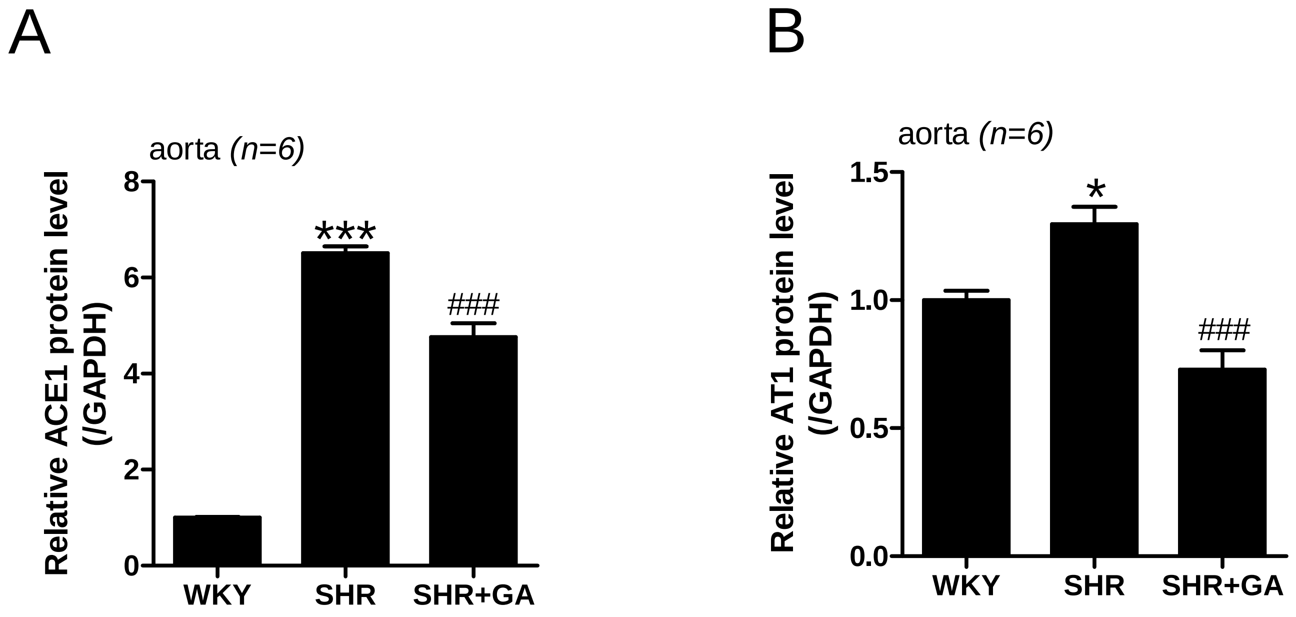

A
B
aorta (n=6)
Relative ACE1 protein level (/GAPDH)
0
2
4
6
8
***
###
WKY
SHR
SHR+GA
aorta (n=6)
Relative AT1 protein level (/GAPDH)
0.0
0.5
1.0
1.5
*
###
WKY
SHR
SHR+GA

Figure S3

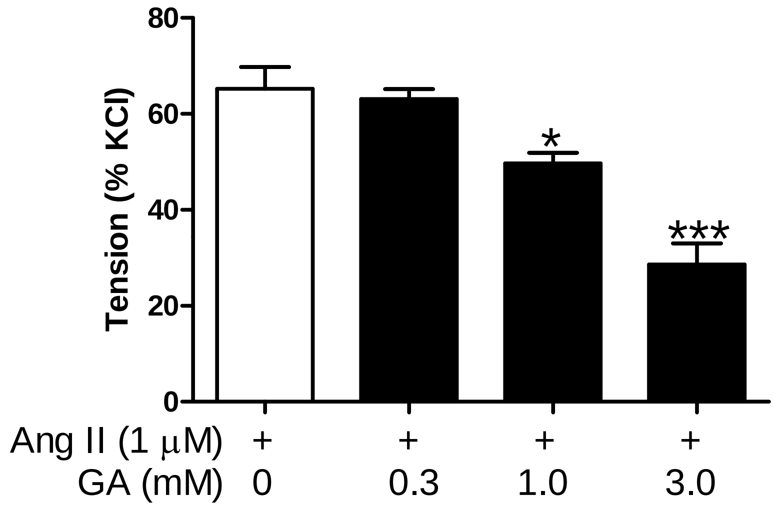

Figure S4

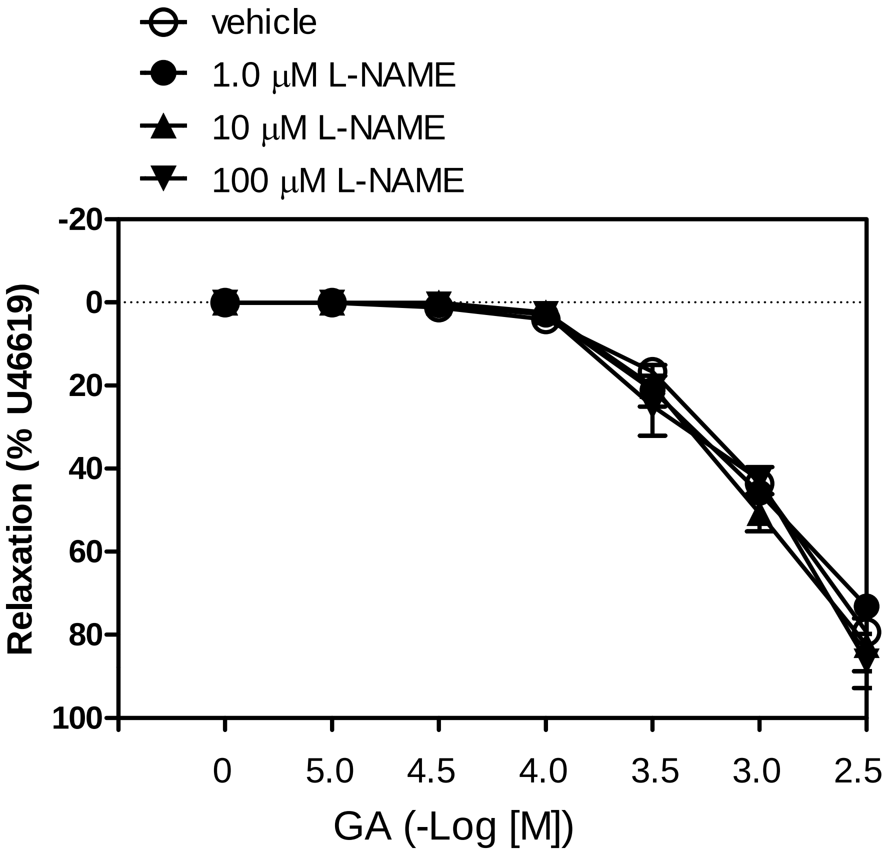

vehicle
1.0 μM L-NAME
10 μM L-NAME
100 μM L-NAME
-20
0
20
40
60
80
100
Relaxation (% U46619)
0
5.0
4.5
4.0
3.5
3.0
2.5
GA (-Log [M])

Figure S5

A

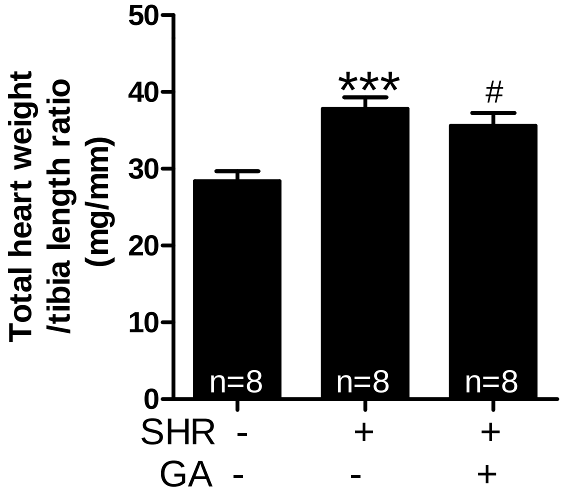
Total heart weight
/tibia length ratio
(mg/mm)
50
40
30
20
10
0
***
#
n=8
n=8
n=8
SHR - + +
GA - - +

B

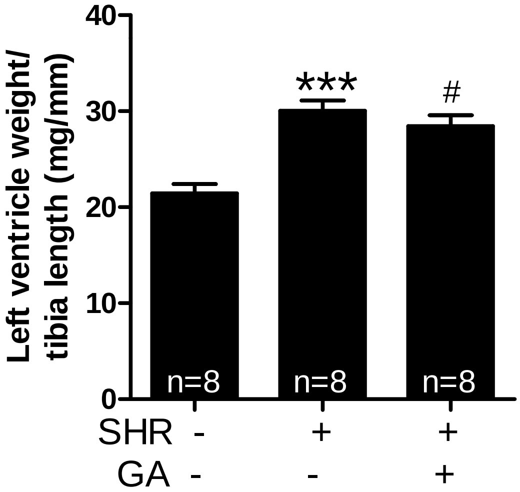
Left ventricle weight/
tibia length (mg/mm)
40
30
20
10
0
***
#
n=8
n=8
n=8
SHR - + +
GA - - +

Figure S6

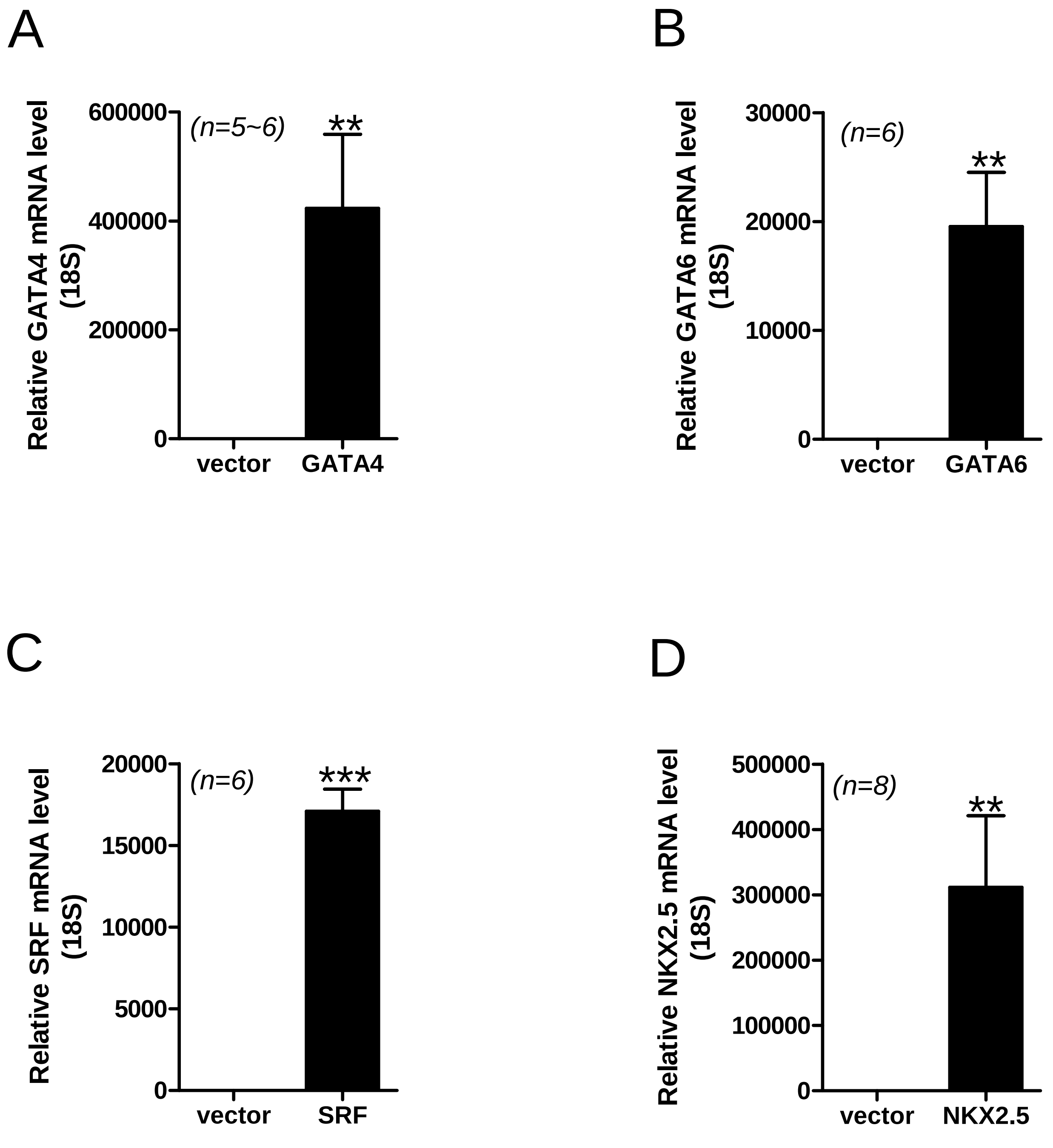
A
Relative GATA4 mRNA level (18S)
600000
400000
200000
0
(n=5~6)
**
vector
GATA4
B
Relative GATA6 mRNA level (18S)
30000
20000
10000
0
(n=6)
**
vector
GATA6
C
Relative SRF mRNA level (18S)
20000
15000
10000
5000
0
(n=6)
***
vector
SRF
D
Relative NKX2.5 mRNA level (18S)
500000
400000
300000
200000
100000
0
(n=8)
**
vector
NKX2.5

Figure S7

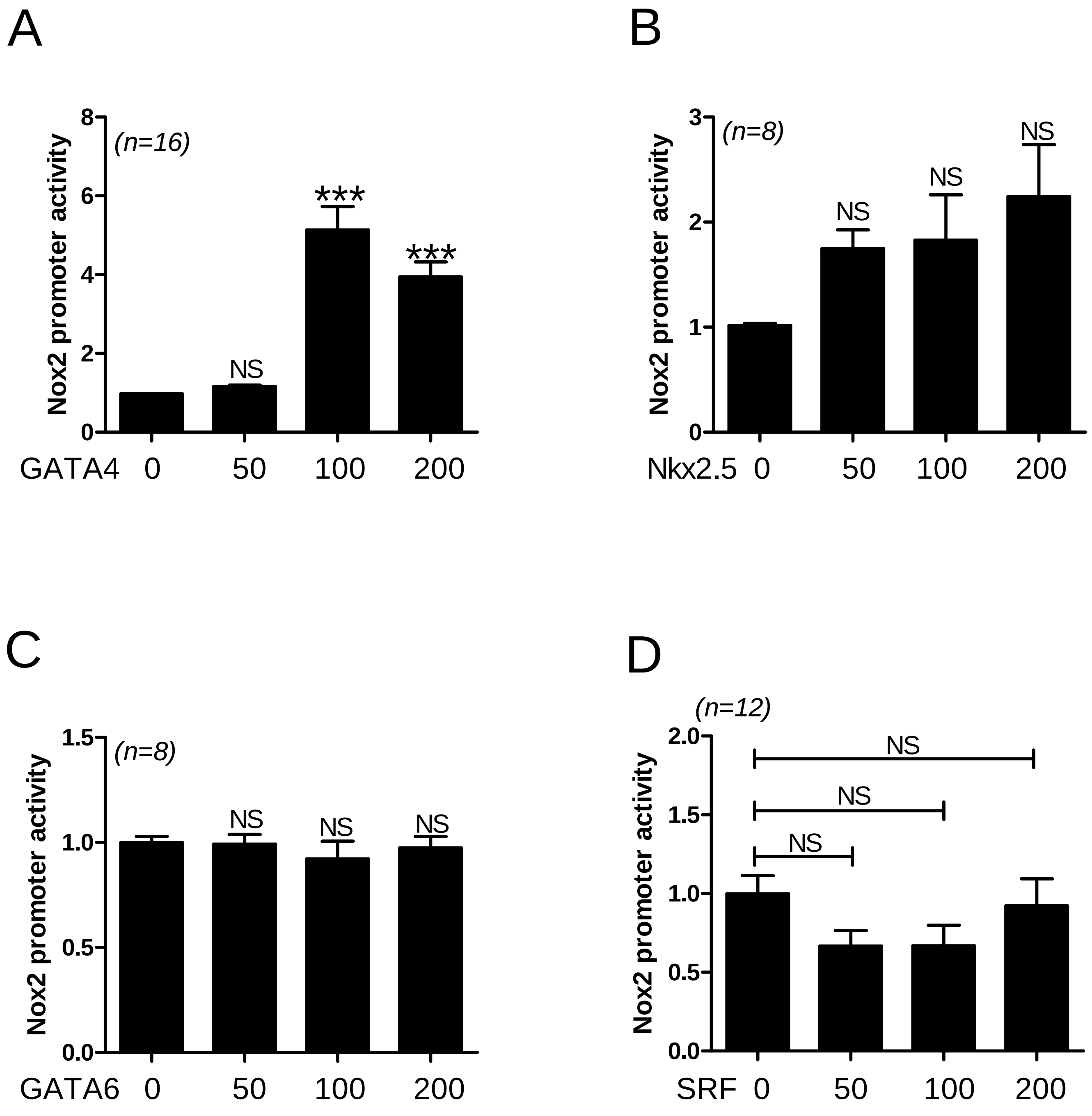
A
Nox2 promoter activity
(n=16)
0
2
4
6
8
NS
***
***
GATA4 0 50 100 200
B
Nox2 promoter activity
(n=8)
0
1
2
3
NS
NS
NS
Nkx2.5 0 50 100 200
C
Nox2 promoter activity
(n=8)
0.0
0.5
1.0
1.5
NS
NS
NS
GATA6 0 50 100 200
D
Nox2 promoter activity
(n=12)
0.0
0.5
1.0
1.5
2.0
NS
NS
NS
SRF 0 50 100 200

Figure S8

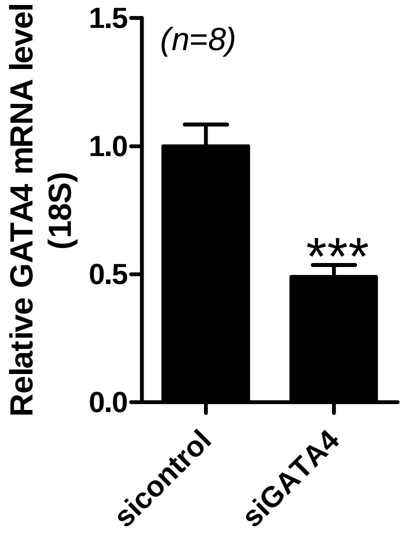

Relative GATA4 mRNA level
(18S)
1.5
1.0
0.5
0.0
(n=8)
***
sicontrol
siGATA4

Figure S9

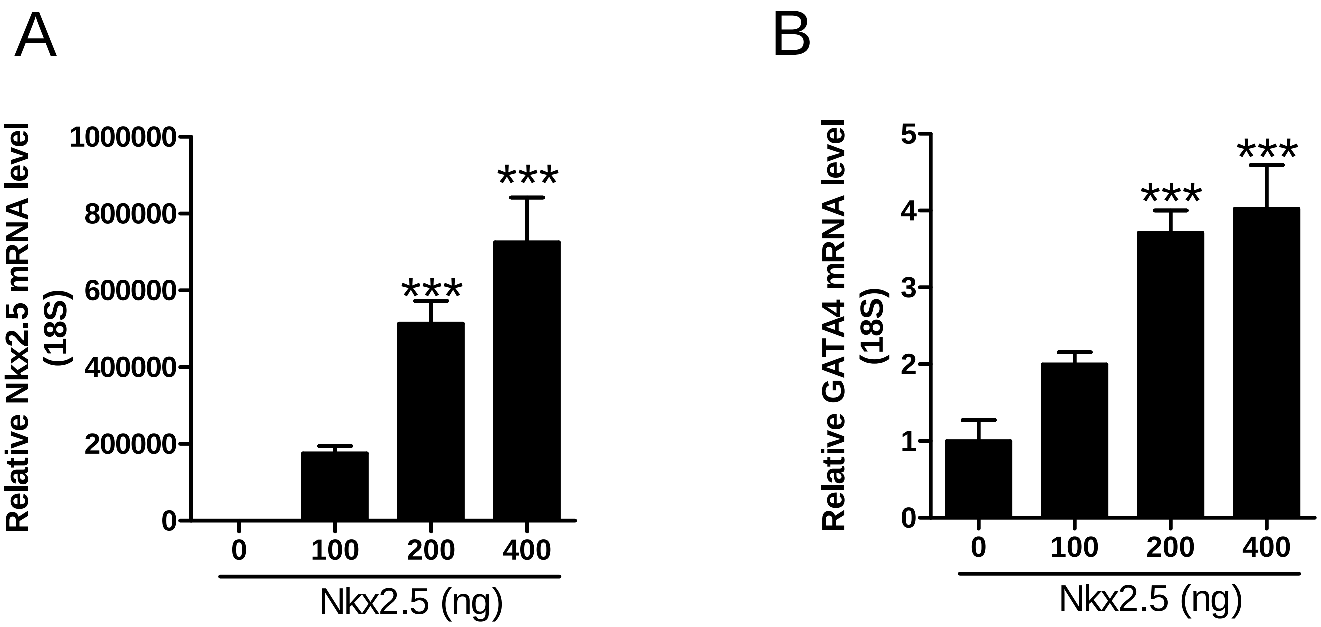
A
B
Relative Nkx2.5 mRNA level
(18S)
1000000
800000
600000
400000
200000
0
***
***
0
100
200
400
Nkx2.5 (ng)
Relative GATA4 mRNA level
(18S)
5
4
3
2
1
0
***
***
0
100
200
400
Nkx2.5 (ng)

Supplementary information : (Figure 1D)

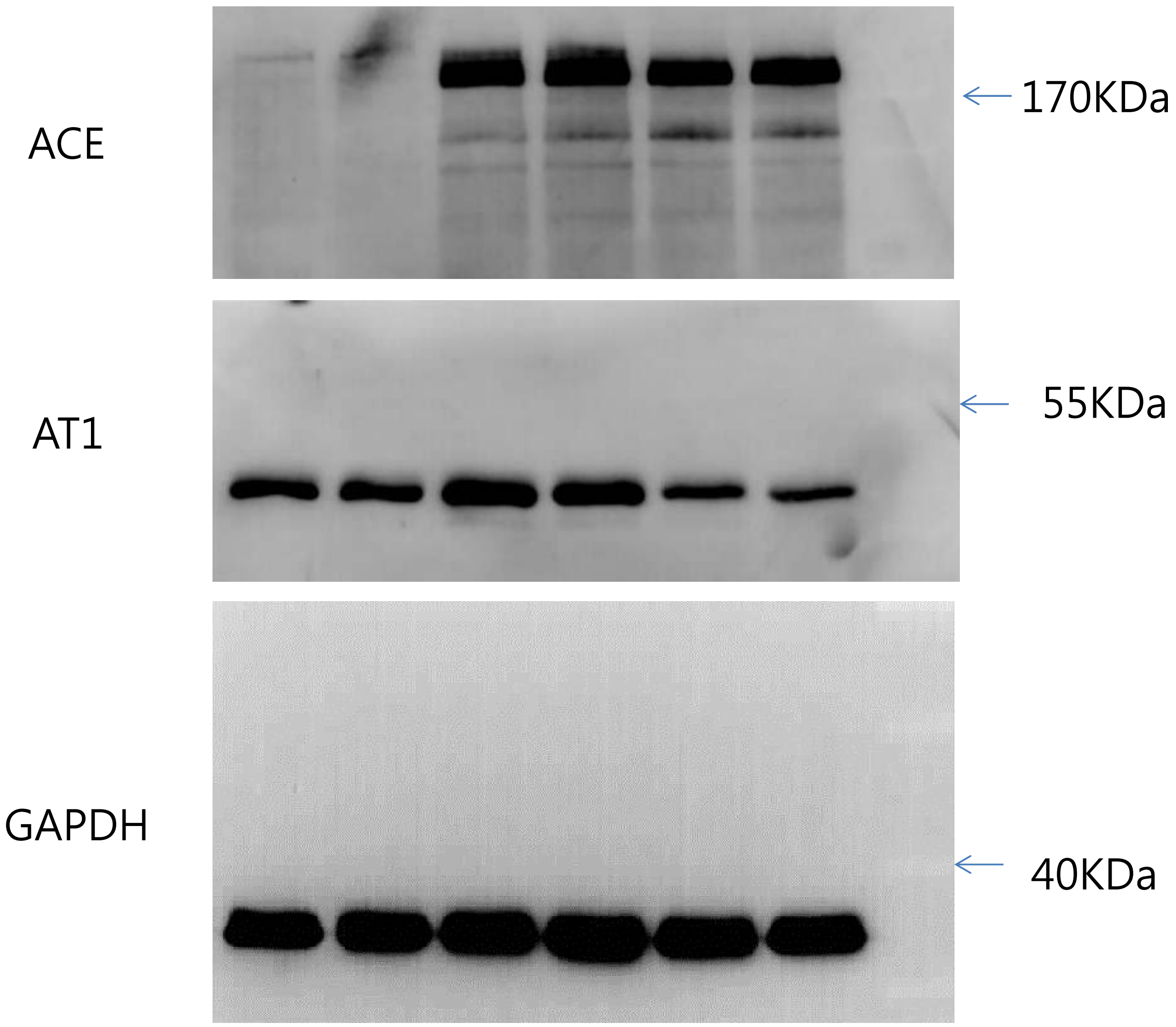

Supplementary information : (Figure 4C)

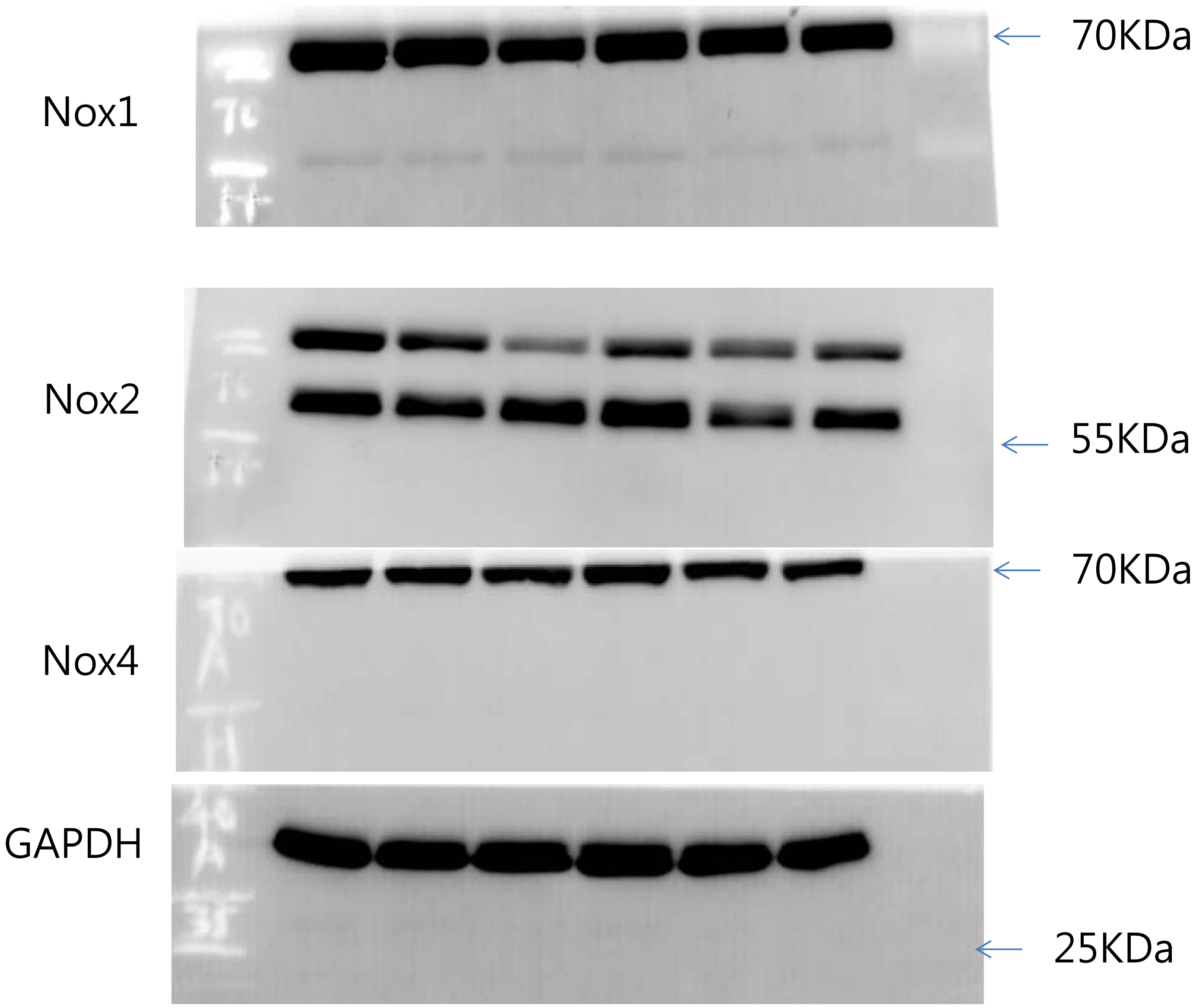

Supplement: Supplementary file 1 — Supplementary figures and information [file 41598_2017_15925_MOESM1_ESM.pdf]
